# Proteomic Dynamics of Ovine Allantoic and Amniotic Fluids During Gestation

**DOI:** 10.1111/rda.70300

**Published:** 2026-08-06

**Authors:** Viviane Maria Codognoto, Rogério Araújo de Almeida Filho, Marcos Gomides Carvalho, Paula Zanin Rates, Flávio Augusto Lourencetti, Laiza Sartori de Camargo, Claudio Willian Delfes de Camargo, Juanita Valentina Guzmán Martínez, Luan Sitó da Silva, Eunice Oba

**Affiliations:** ^1^ Department of Veterinary Surgery and Animal Reproduction, Faculty of Veterinary Medicine and Animal Science UNESP Botucatu Brazil

**Keywords:** fetal development, mass spectrometry, maternal–foetal communication, *Ovis aries*, uterine fluids

## Abstract

Gestational success in ruminants depends on the maintenance of a dynamic maternal–foetal microenvironment, resulting from complex interactions among the conceptus, extraembryonic membranes, maternal tissues, and gestational fluids. This study performed a comparative and longitudinal proteomic characterization of allantoic and amniotic fluids throughout pregnancy in sheep, aiming to elucidate the molecular mechanisms involved in maternal–foetal communication and embryonic development. Samples of allantoic and amniotic fluids aseptically collected from extraembryonic compartments after uterine opening following animal slaughter and allocated into three gestational groups according to foetal development: early gestation (0–50 days; *n* = 6), mid gestation (50–100 days; *n* = 3) and late gestation (100–150 days; *n* = 5). After centrifugation and protein quantification using the Bradford method, standardized aliquots were subjected to in‐solution protein digestion and analysed by nano‐LC–ESI‐Q‐TOF mass spectrometry. A total of 201 proteins were identified in the allantoic fluid and 159 proteins in the amniotic fluid, with five proteins showing significant differences across gestational stages. In the allantoic fluid, differentially abundant proteins included alpha‐2‐macroglobulin, gelsolin, basement membrane–specific heparan sulphate proteoglycan core protein and alpha‐globin chain. In the amniotic fluid, changes were observed in peroxiredoxin‐2, mucin‐5 AC, fetuin‐B, cystatin domain–containing protein and globin domain–containing protein. Multivariate analyses revealed progressive remodelling of the foetal extraembryonic microenvironment, associated with proteolytic control, tissue remodelling, redox homeostasis, epithelial protection and metabolic processes. Collectively, these findings reinforce the active role of foetal extraembryonic fluids in gestational physiology and highlight their potential as sources of biomarkers associated with reproductive success in sheep.

## 1 Introduction

Gestational success is directly associated with the maintenance of a regulated and highly dynamic maternal–foetal microenvironment, in which interactions between the maternal endometrium, the conceptus and extraembryonic membranes drive essential processes of implantation, histotroph synthesis and molecular signalling required for embryonic development and the continuation of pregnancy in ruminants (Bazer, Kim, et al. 2012). In this context, extraembryonic gestational fluids, including allantoic and amniotic fluids, play a central role as biochemical mediators of maternal–foetal communication, reflecting both the metabolic activity of the conceptus and physiological exchanges occurring at the maternal–foetal interface throughout pregnancy in ruminants (Bazer et al. 2009; Spencer et al. 2017).

Among these fluids, amniotic fluid, which was previously considered merely a physical protective barrier, is now recognized as a biologically active fluid, playing a fundamental role in foetal pulmonary, musculoskeletal and immunological development (Dickson et al. 1990; Riding et al. 2008; Underwood et al. 2005), in addition to acting as a mediator of molecular signalling between the conceptus and maternal tissues at the maternal–foetal interface (Riding et al. 2008). Its protein composition results from the integration of foetal secretions derived from the respiratory system, skin, urinary tract, extraembryonic membranes and possible maternal‐derived components transferred through maternal–foetal exchange mechanisms. Thus, amniotic fluid acts as a dynamic molecular record, reflecting foetal physiological status, health and degree of maturation (Underwood et al. 2005). Similarly, in species with an epitheliochorial placenta, such as ruminants, allantoic fluid constitutes an extraembryonic compartment and plays essential roles in nutrient transport and redistribution, the accumulation and elimination of foetal metabolites, as well as in the modulation of the immunological environment during the early and mid stages of pregnancy (Bazer, Kim, et al. 2012). The functional interaction between allantoic and amniotic fluids contributes in an integrated manner to the maintenance of gestation homeostasis, maternal–foetal metabolic adaptation, support of foetal growth and foetal maturation, highlighting the importance of these fluids within the extraembryonic gestational microenvironment (Spencer et al. 2017).

Within this framework, comparative proteomic analysis of extraembryonic gestational fluids in animal models emerges as a strategic approach to elucidate the complex maternal–foetal physiology and the molecular mechanisms underlying gestational success (Forde et al. 2014; Riding et al. 2008). This approach has enabled the identification of proteins associated with key foetal developmental processes, including maternal–foetal metabolic adaptation and physiological processes occurring within the maternal–foetal interface, as well as the characterization of dynamic proteomic variations throughout gestation, reflecting changes in foetal growth, pulmonary maturation and the activation of immunological and antioxidant pathways (Boy et al. 2019; Forde et al. 2014; Riding et al. 2008).

These studies reinforce the potential of proteomics applied to amniotic and allantoic fluids as a valuable tool for investigating the molecular mechanisms underlying maternal–foetal physiology in animal models, as well as for identifying potential biomarkers associated with the establishment and maintenance of pregnancy. In this regard, the aim of the present study is to characterize and compare the proteomic dynamics of amniotic and allantoic fluids in sheep during the early, mid and late thirds of gestation, in order to elucidate the molecular mechanisms involved in maternal–foetal communication, embryonic development and the maintenance of the maternal–foetal and extraembryonic gestational microenvironment.

## 2 Materials and Methods

### 2.1 Reagents

All reagents used were of high purity and were obtained from Sigma‐Aldrich (St. Louis, Missouri, USA), GE Healthcare Life Sciences (São Paulo, SP, Brazil), Waters Corporation (Barueri, SP, Brazil), Bio‐Rad Laboratories (Hercules, California, USA) and Thermo Fisher Scientific (São Paulo, SP, Brazil) unless otherwise specified.

### 2.2 Ethical Aspects

The study was conducted with the approval of the Ethics Committee on the Use of Animals (CEUA), under protocol no. 0112/2019, and followed the ethical guidelines and recommendations of the National Council for the Control of Animal Experimentation (CONCEA).

### 2.3 Sample Collection

Twenty‐four uterine samples from pregnant ewes were collected at different gestational ages from slaughterhouses in the state of São Paulo, Brazil. Samples were categorized into early gestation (0–50 days, *n* = 11), mid‐gestation (50–100 days, *n* = 8) and late gestation (100–150 days, *n* = 5). Allantoic and amniotic fluids from each uterus were collected using sterile syringes and stored in sterile tubes. However, during sample collection, some samples were lost and during proteomic analysis, three samples did not yield valid results, resulting in a reduced experimental n for the amniotic fluid group: early gestation (*n* = 6), mid‐gestation (*n* = 3) and late gestation (*n* = 5). In contrast, the experimental n for the allantoic fluid group remained unchanged (early gestation *n* = 11, mid‐gestation *n* = 8 and late gestation *n* = 5). Each fetus was weighed, the foetal crown–rump length was measured and coat development was assessed. In the laboratory, samples of allantoic and amniotic fluids were centrifuged at 15,000 × g for 30 min at 4°C to remove cellular debris and subsequently stored at −80°C.

### 2.4 Proteomic Analysis

The total protein concentration of allantoic and amniotic fluids was determined using a spectrophotometer (Ultrospec 2000, Pharmacia Biotech, Ultrospec 2000 UV/VIS Spectrophotometer, Uppsala, Sweden) according to the Bradford method (B6916, Sigma‐Aldrich, St. Louis, MO, USA). Protein concentrations were expressed in μg/μL based on a standard curve generated using known concentrations of bovine serum albumin.

An aliquot (50 μg) of protein from each sample was subjected to electrophoresis on a 10% polyacrylamide gel (SDS‐PAGE). After sample entry into the resolving gel, electrophoresis was interrupted and the gel was stained with colloidal Coomassie blue (Luo et al. 2006; Neuhoff et al. 1985). Stained bands were excised and subjected to in‐gel tryptic digestion.

Protein digestion was performed using the method described by Carvalho et al. (2021), with minor modifications. Briefly, gel pieces were destained with 50% methanol and 2.5% acetic acid in ultrapure water, followed by dehydration with 100% acetonitrile. Disulphide bonds were reduced with dithiothreitol (DTT) and alkylated with iodoacetamide. Samples were digested overnight with trypsin (20 ng/μL) at a 1:50 (trypsin/substrate) ratio (Trypsin, Promega, Cat. No. V511, São Paulo, SP, Brazil). Peptides were extracted using 5% formic acid. The resulting samples were concentrated using a SpeedVac system (SPD1010 Integrated SpeedVac Systems, Thermo Fisher Scientific Inc., Waltham, MA, USA) and stored at −80°C until mass spectrometry analysis.

Prior to analysis, samples were thawed, diluted with 0.1% formic acid to a final concentration of 0.7 μg protein/μL, homogenized and centrifuged at 1100 × g for 5 min. For mass spectrometry analysis, a 4.5 μL aliquot was separated on a C18 column (100 μm × 100 mm) using reverse‐phase nano ultra‐high‐performance liquid chromatography (RP‐nano UPLC; Waters nanoACQUITY UPLC, Waters Corporation, Milford, MA, USA) coupled to a quadrupole time‐of‐flight mass spectrometer (Q‐TOF) (Micromass Q‐ToF PREMIER, Waters Corporation, Milford, MA, USA) equipped with a nano‐electrospray ionization source, operating at a flow rate of 0.600 μL/min. A linear gradient of acetonitrile (2%–90%) in 0.1% formic acid was applied over 45 min. The nano‐electrospray voltage was set to 3.5 kV, the cone voltage to 30 V and the source temperature to 100°C. The instrument was operated in top‐three mode, in which a full MS scan was acquired followed by MS/MS fragmentation of the three most intense precursor ions.

Database search parameters included trypsin as the protease, allowing up to one missed cleavage; carbamidomethylation of cysteine as a fixed modification and oxidation of methionine as a variable modification; a mass tolerance of 1 Da for both precursor (MS) and fragment (MS/MS) ions; and monoisotopic mass selection. Spectra were acquired using MassLynx software v.4.1 (Waters Corporation, Milford, MA, USA), and raw data files were converted to peak list format (.mgf) without adding scans. The resulting files were searched against the UniProt/Swiss‐Prot database (http://www.uniprot.org/) for the *Ovis aries* taxonomy using Mascot version 2.3.02 and Mascot Distiller MDRO version 2.4.0.0 (Matrix Science Inc., Boston, MA, USA). Relative protein abundance within each sample was estimated by calculating the exponentially modified protein abundance index (emPAI) using Mascot Distiller software.

### 2.5 Data Analysis

Data normalization considered only proteins detected in at least 50% of the samples within each group. To minimize the effects of outliers, the emPAI value of each sample was divided by the sum of emPAI values of all samples within the respective groups. The resulting normalized values were used for subsequent statistical analyses.

The data were subjected to non‐hierarchical cluster analysis using MetaboAnalyst 6.0 software (https://www.metaboanalyst.ca/). To confirm group separation and to assess the influence of outliers on protein abundance, multivariate analysis—specifically principal component analysis (PCA)—was applied to describe sample variation within the score matrix. A heatmap was generated to visually compare protein abundance across groups. Univariate analysis (ANOVA) was used to identify differences among groups, followed by Fisher's least significant difference (LSD) test for multiple comparisons. Results were considered statistically significant when the false discovery rate (FDR) was < 0.05.

Gene ontology annotation for the differentially expressed proteins in the clusters was obtained from ShinyGO 0.85.1 (https://bioinformatics.sdstate.edu/go/) using the categories molecular function, biological process and cellular component.

The mass spectrometry proteomics data were deposited in the Mendeley Data (https://data.mendeley.com/) repository with the data set identifier DOI: 10.17632/k3bvmwv3jw.1.

## 3 Results

**FIGURE 1:**
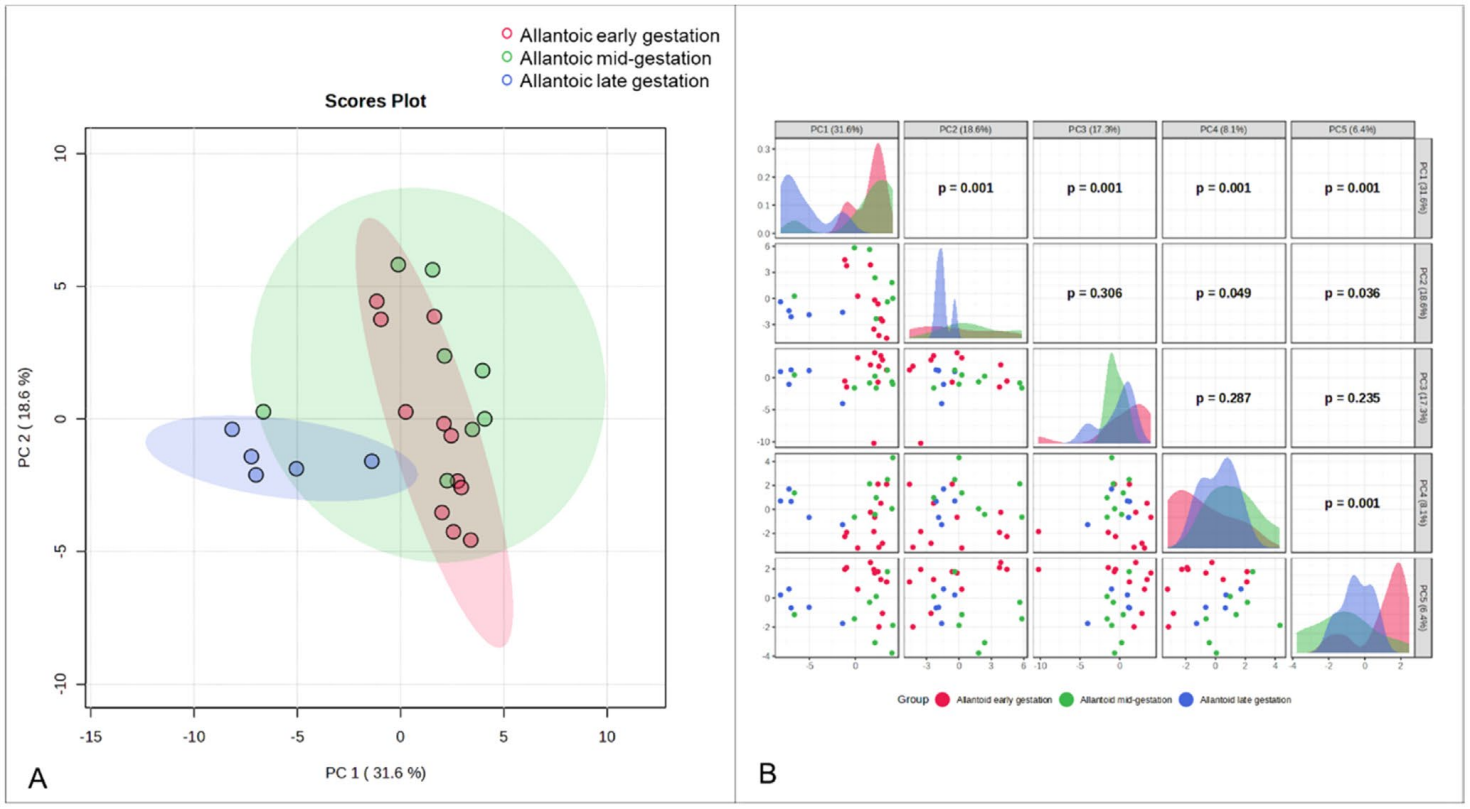
Results of multivariate cluster analysis. (A) Principal component analysis (PCA) of allantoic fluid proteins from early, mid and late gestation in ovine showing variation among groups (PC1 + PC2 = 50.2%). (B) PERMANOVA results: *F* = 8.3299, *R* ^2^ = 0.44238, *p* = 0.001 (based on 999 permutations).

Based on the principal component analysis (PCA) and dendrogram (Figure 1), the results demonstrated a clear distinction in the protein profile of allantoic fluid from ewes at the early, mid and late stages of gestation, as the combined variance explained by PC1 (31.6%) and PC2 (18.6%) accounted for 50.2% of the total variance.

**FIGURE 2:**
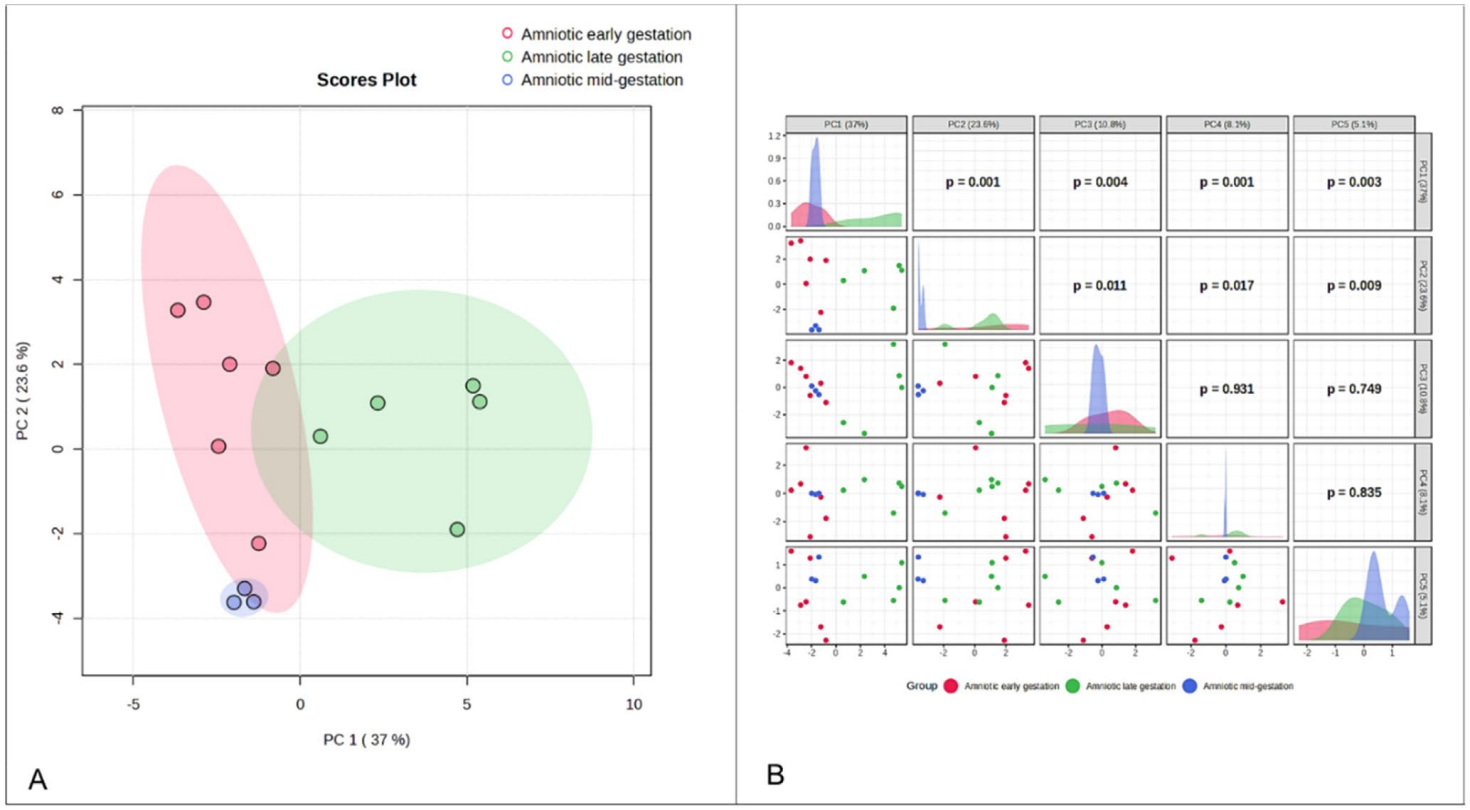
Results of multivariate cluster analysis. (A) Principal component analysis (PCA) of allantoic fluid proteins from early, mid and late gestation in ovine showing variation among groups (PC1 + PC2 = 60.6%). (B) PERMANOVA: *F*‐value = 15.598; R‐squared = 0.73932; *p*‐value (based on 999 permutations) = 0.001.

Similar results were observed in the PCA and dendrogram analyses of amniotic fluid, which demonstrated a clear distinction in the protein profiles of ewes at the early, mid and late stages of gestation, as the combined variance explained by PC1 (37.0%) and PC2 (23.6%) accounted for 60.6% of the total variance (Figure 2). This value exceeded 50%, indicating significant differences among gestational stages in both fluids.

**FIGURE 3:**
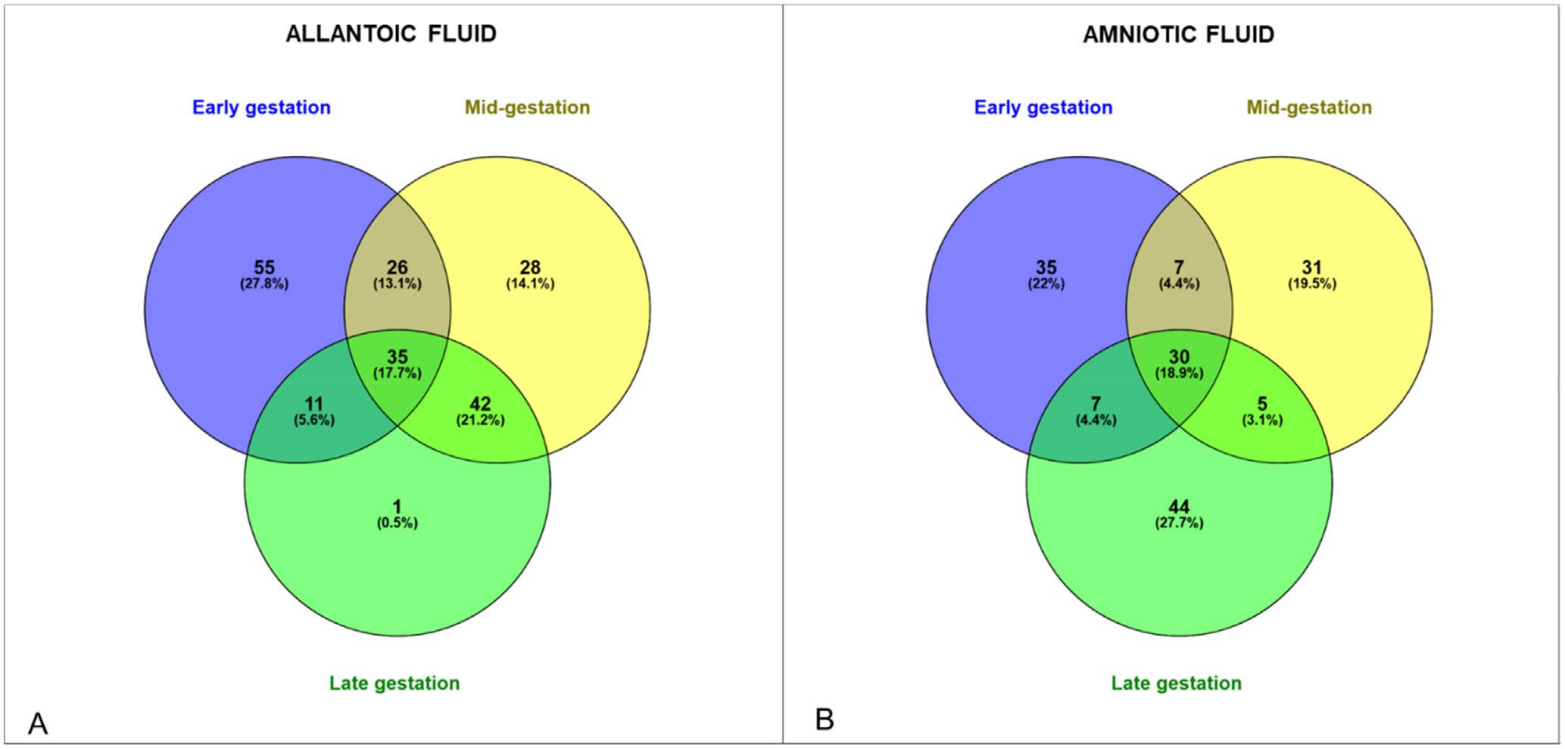
Venn diagram illustrating the total number of proteins identified in allantoic and amniotic fluids during early, mid and late gestation.

A total of 201 proteins were identified in allantoic fluid and 159 proteins in amniotic fluid across the early, mid and late stages of gestation in sheep. Among these, 35 proteins were common to all three gestational stages in allantoic fluid, whereas 30 proteins were common in amniotic fluid (Figure 3).

**FIGURE 4:**
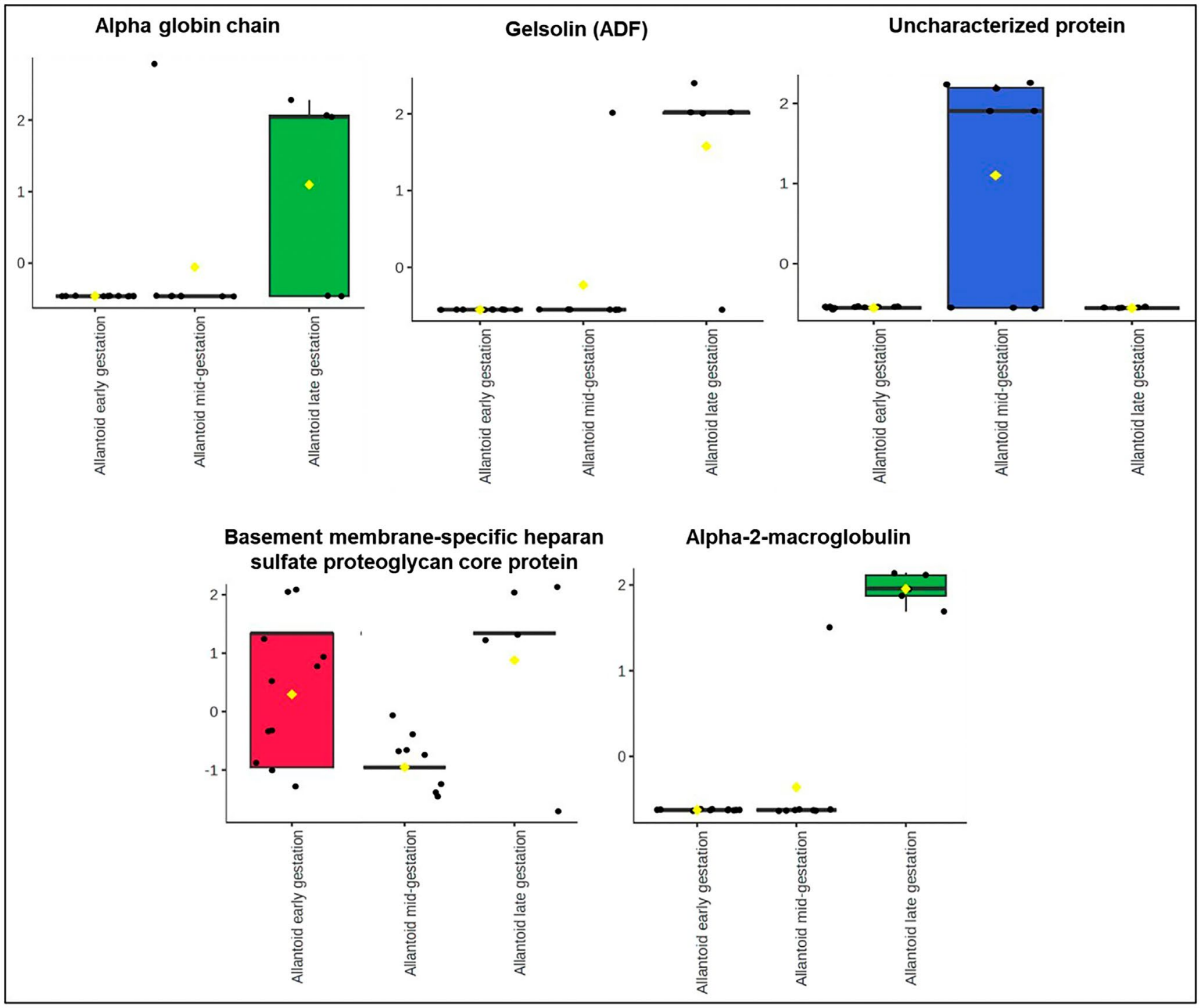
Proteins showing significant differences (*p* < 0.01) among the allantoic fluid groups at early, mid and late gestation.

**FIGURE 5:**
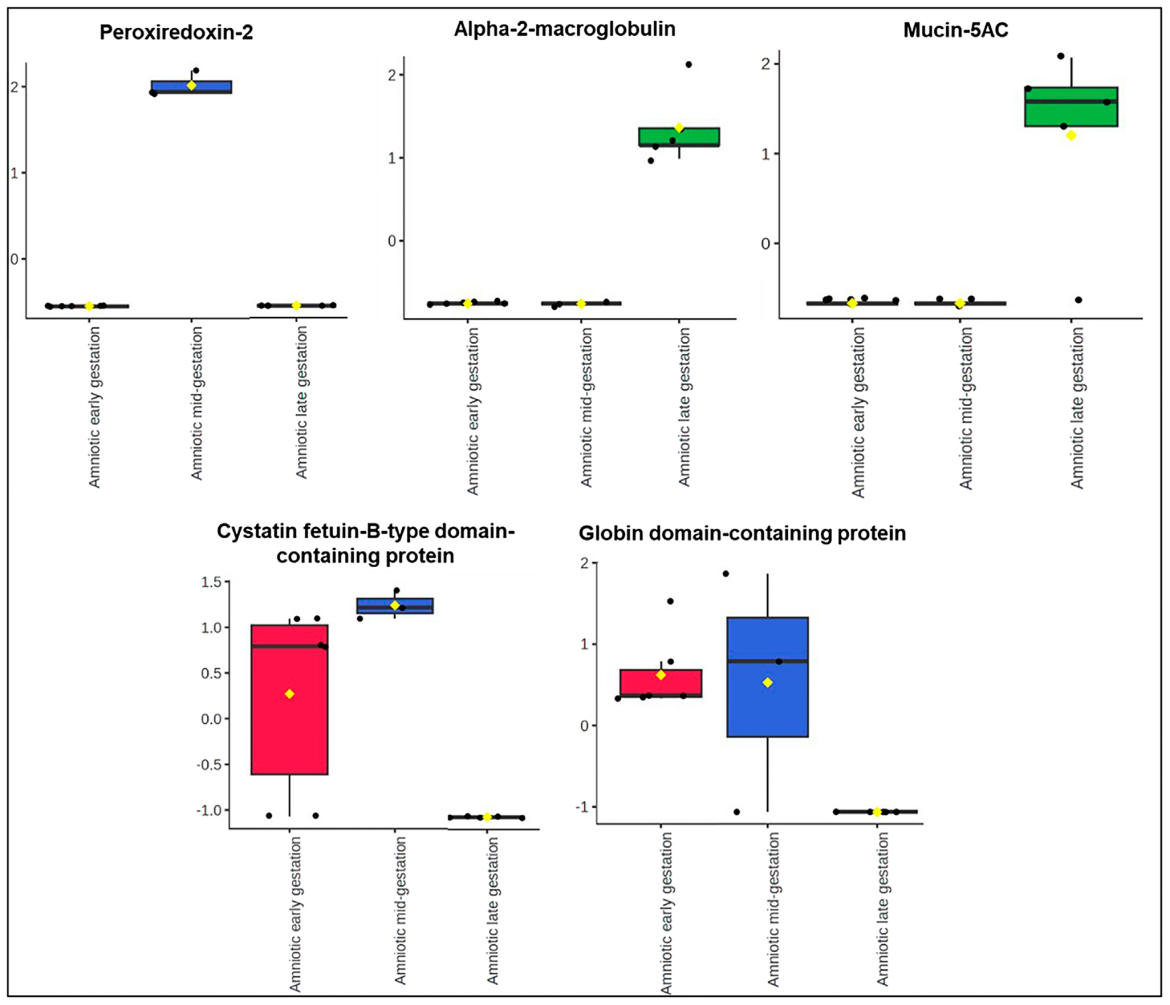
Proteins showing significant differences (*p* < 0.01) among the amniotic fluid groups at early, mid and late gestation.

Among the identified proteins, five showed significant differences across the evaluated gestational stages. In allantoic fluid, the differentially abundant proteins were alpha‐2‐macroglobulin, gelsolin (ADF), an uncharacterized protein, basement membrane–specific heparan sulphate proteoglycan core protein and alpha globin chain (Figure 4). In amniotic fluid, the proteins showing significant differences were peroxiredoxin‐2, alpha‐2‐macroglobulin, mucin‐5 AC, cystatin fetuin‐B‐type domain–containing protein and globin domain–containing protein (Figure 5).

Gene Ontology analysis of all proteins was performed using ShinyGO 0.85.1 (https://bioinformatics.sdstate.edu/go) to identify the main molecular functions, biological processes and cellular components. The predominant molecular functions identified in the allantoic fluid were haptoglobin binding and oxygen carrier activity during early gestation, whereas haptoglobin binding and peroxiredoxin activity predominated during mid and late gestation. Regarding biological processes, oxygen transport and regulation of monoatomic anion transport were the most enriched processes during early and mid‐gestation, while oxygen transport and gas transport predominated during late gestation. For the cellular component category, the haptoglobin–haemoglobin complex was the most enriched component across all gestational stages (Figure S1). The predominant molecular functions identified in the amniotic fluid were haptoglobin binding and peroxiredoxin activity during early and mid‐gestation, whereas haptoglobin binding and oxygen carrier activity predominated during late gestation. Regarding biological processes, oxygen transport and gas transport were the most enriched processes across all gestational stages. For the cellular component category, the haptoglobin–haemoglobin complex was the most enriched component throughout all phases of gestation (Figure S2).

## 4 Discussion

The present study represents the first investigation to integratively examine the proteomic profiles of allantoic and amniotic fluids throughout gestation in sheep, thereby filling an important gap in current knowledge of maternal–foetal physiology in this species. Although studies in ruminants, particularly in cattle, have demonstrated that gestational fluids constitute biologically active extraembryonic compartments (Forde et al. 2014; Riding et al. 2008), information regarding the specific molecular composition and biological functions of these fluids in sheep remains limited. In this context, the proteomic characterization of allantoic and amniotic fluids emerges as a strategic approach to elucidate the mechanisms regulating foetal development, the adaptation of extraembryonic membranes and the maintenance of the maternal–foetal and extraembryonic gestational microenvironment across different stages of pregnancy. Accordingly, the analysis presented in this study provides a foundation for advancing the understanding of gestational physiology in sheep, enabling deeper insight into maternal–foetal interactions and the functional role of extraembryonic foetal fluids in the reproductive success of the species.

Consistently, multivariate analyses revealed a clear pattern of sample clustering, as demonstrated by principal component analysis (PCA), with distinct differences in protein abundance among gestational stages in both allantoic and amniotic fluids, indicating compartment‐specific molecular profiles. These findings provide proteomic evidence that the extraembryonic foetal fluid microenvironment undergoes a continuous and highly regulated remodelling process throughout gestation, reflecting functional adaptations associated with foetal development (Brace and Cheung 2014). In ruminants, previous studies have shown that gestational and extraembryonic fluids constitute dynamic compartments whose molecular composition mirrors the progressive and coordinated changes that occur during the development and maintenance of pregnancy (Codognoto et al. 2024; Forde et al. 2014; Riding et al. 2008; Spencer et al. 2017).

In this context, amniotic fluid, due to its more direct and continuous interaction with the foetus, has been recognized as an important reflector of processes associated with the metabolic demands of the developing foetus, including pathways related to protein synthesis, cellular growth and tissue differentiation (Bazer, Song, et al. 2012; Kwon et al. 2003). In addition, amniotic fluid shows potential applicability as a diagnostic tool in pathological conditions of pregnancy, including placentitis (Loux and Ball 2018), further reinforcing its role as a biologically active and informative compartment of the maternal–foetal and extraembryonic physiological state (Bazer, Song, et al. 2012; Kwon et al. 2003; Loux and Ball 2018). In contrast, allantoic fluid plays a central role in metabolic homeostasis and in metabolite exchange between the foetus and the extraembryonic membranes (Kwon et al. 2003; Riding et al. 2008). The consistent separation of gestational stages supports the existence of phase‐specific molecular signatures, likely regulated by sequential transcriptional programs in the foetus and its associated tissues, which coordinate embryonic development and adaptation of the extraembryonic gestational microenvironment (Bazer, Song, et al. 2012; Riding et al. 2008; Spencer et al. 2016). Collectively, these aspects support the notion that the protein composition of foetal fluids reflects both conserved essential functions and gestation stage–specific adaptations in ruminants (Boy et al. 2019; Codognoto et al. 2024; Riding et al. 2008).

Among the differentially expressed proteins, alpha‐2‐macroglobulin stood out as significantly altered in both fluids during the final third of gestation. Alpha‐2‐macroglobulin is a multifunctional plasma glycoprotein widely recognized for its protease‐inhibitory activity, which neutralizes proteolytic enzymes involved in inflammatory processes and tissue remodelling. In addition, alpha‐2‐macroglobulin plays a central role in regulating inflammatory and immune responses by functioning as a carrier molecule and modulator of the bioavailability of cytokines, growth factors and peptide hormones, thereby influencing their stability, half‐life and interactions with cellular receptors (Garcia‐Ferrer et al. 2017; Rehman et al. 2013; Zhai et al. 2025). This protein has previously been reported in the allantoic and amniotic fluids of cows, mares and buffaloes at different stages of gestation, with higher abundance in allantoic fluid (Boy et al. 2019; Loux and Ball 2018; Riding et al. 2008), findings consistent with ours. The temporal regulation of alpha‐2‐macroglobulin throughout gestation may reflect physiological adaptations associated with the regulation of proteolytic activity within the extraembryonic foetal microenvironment, including the control of matrix metalloproteinases and other proteases involved in placental and foetal membrane remodelling. This regulatory balance may contribute to the maintenance of extraembryonic membrane integrity and local homeostasis during advanced stages of pregnancy (Barrett and Starkey 1973; Sottrup‐Jensen 1989). This interpretation is supported by studies in humans showing that altered alpha‐2‐macroglobulin levels are associated with pregnancy complications, such as preeclampsia, a condition characterized by dysregulated trophoblast invasion and vascular remodelling (Wang et al. 2023). Therefore, our results indicate a potential involvement of alpha‐2‐macroglobulin in proteostasis at the maternal–foetal interface rather than a direct biomarker role in the ovine model.

Higher abundance of the alpha globin chain and gelsolin was observed in allantoic fluid during the final third of gestation, which may reflect physiological adaptations associated with intensified haematopoiesis and increased metabolic demands of the foetus in ruminants. As a structural component of haemoglobin, the alpha‐globin chain is directly involved in oxygen transport and foetal haematological development (Voon and Vadolas 2008), processes that become more pronounced during advanced stages of pregnancy. Considering the close association of allantoic fluid with foetal circulation and the urinary system, its proteomic composition may indirectly reflect changes associated with foetal erythropoiesis and increased oxygen demand rather than a direct measurement of erythrocyte mass during this period. The presence and modulation of gelsolin in foetal fluids suggest its involvement in cellular and tissue remodelling during foetal development. Gelsolin is a key protein in the dynamic regulation of actin filaments, mediating actin severing, capping and reorganization in a calcium‐dependent manner and is essential for cell migration, morphogenesis and structural adaptation of developing tissues (Guo et al. 2018; Lader et al. 1999). In gestational contexts, the detection of gelsolin in biological fluids has been associated with processes involving high cellular turnover and the intense tissue reorganization characteristic of the placenta and foetal membranes. Additionally, gelsolin may exert a protective role by scavenging extracellular actin released during physiological processes of cellular renewal, thereby contributing to the maintenance of homeostasis in the extraembryonic foetal microenvironment homeostasis (Garg et al. 2018; Piktel et al. 2018; Sezen et al. 2009).

The last protein showing higher abundance in allantoic fluid was the basement membrane–specific heparan sulphate proteoglycan core protein, an essential basement membrane proteoglycan involved in extracellular matrix organization, regulation of tissue permeability and modulation of growth factor bioavailability, thereby playing a central role in angiogenesis, morphogenesis and vascular development (Dreyfuss et al. 2009; Iozzo 2005). In ruminants, marked placental expansion and foetal growth require continuous remodelling of the extraembryonic membranes and the associated vascular system, processes in which basement membrane components exert fundamental structural and regulatory functions (Bazer et al. 2009; Spencer et al. 2016). Considering the close association of allantoic fluid with foetal circulation and allantoic tissues, the presence of this proteoglycan in the fluid may reflect ongoing extracellular matrix remodelling and basement membrane turnover rather than a direct structural incorporation into the fluid compartment associated with foetal development throughout the extraembryonic gestational microenvironment.

Peroxiredoxin‐2 was reported in the present study at higher abundance in amniotic fluid during the mid‐gestation period in sheep. This protein is a cytosolic antioxidant enzyme that is highly efficient in reducing peroxides, particularly hydrogen peroxide, thereby contributing to the control of oxidative stress and the maintenance of cellular redox homeostasis (Rhee et al. 2012). During pregnancy, the progressive increase in foetal metabolic activity and tissue oxygenation promotes the generation of reactive oxygen species, underscoring the importance of antioxidant systems in foetal fluids (Myatt and Cui 2004). In ruminants, studies have shown that amniotic fluid serves not only as a physical protective medium but also as a biologically active extraembryonic compartment, reflecting metabolic, immunological and antioxidant processes associated with foetal development (Dickson et al. 1990; Riding et al. 2008). In this context, the presence of peroxiredoxin‐2 in amniotic fluid suggests a potential contribution to antioxidant defence mechanisms that support redox balance within the extraembryonic foetal microenvironment throughout gestation.

The detection of mucin‐5 AC in sheep amniotic fluid during the final third of gestation suggests a role in the mechanisms involved in protecting and maintaining the integrity of the extraembryonic foetal microenvironment. Mucin‐5 AC is a gel‐forming mucin widely recognized for its role in mucosal barrier formation, contributing to lubrication, mechanical protection and defence against pathogenic agents by modulating viscosity and entrapping microorganisms and particles (McGuckin et al. 2011; Thornton et al. 2008). In gestational contexts, the presence of mucins in foetal fluids has been associated with the protection of foetal epithelial surfaces, including the developing skin and respiratory tract, as well as with contributions to innate immune defence within the maternal–foetal interface and the extraembryonic environment (Rose and Voynow 2006; Thornton et al. 2008; Underwood et al. 2005). Thus, the identification of mucin‐5 AC in ovine amniotic fluid may indicate a potential role in epithelial protection and in maintaining homeostasis within the extraembryonic foetal environment, particularly during stages of gestation characterized by increased foetal exposure to mechanical and immunological stimuli.

The cystatin fetuin‐B–type domain–containing protein detected at higher abundance in sheep amniotic fluid suggests its involvement in mechanisms potentially related to the regulation of proteolytic activity within the extraembryonic foetal microenvironment. Proteins belonging to the cystatin family act as inhibitors of cysteine proteases and play a fundamental role in regulating protein turnover, protecting foetal tissues and modulating inflammatory processes associated with development (Turk et al. 2008). Complementarily, fetuin‐B has been described as a glycoprotein involved in proteolytic homeostasis and protection against excessive protease activity in reproductive contexts, including pregnancy (Dietzel et al. 2013, 2016). This protein has been reported in allantoic fluid of cows (Riding et al. 2008) and in amniotic fluid of buffaloes (Boy et al. 2019). In ruminants, the balance between proteases and their inhibitors is essential for maintaining the integrity of extraembryonic membranes and for proper foetal development, particularly during periods of intense growth and tissue remodelling within the maternal–foetal and extraembryonic interface (Spencer et al. 2016). Thus, the presence of this protein in amniotic fluid reinforces the concept of this compartment as a biologically active medium that may contribute to the maintenance of proteolytic balance and support foetal development within the extraembryonic gestational environment in sheep.

The presence of a globin domain–containing protein in the amniotic fluid of sheep suggests a role associated with oxygen homeostasis and the regulation of redox balance within the extraembryonic foetal microenvironment. Proteins containing globin domains are recognized for their capacity to reversibly bind oxygen and to provide cellular protection against fluctuations in O_2_ availability and oxidative stress (Burmester and Hankeln 2014). In gestational contexts, the detection of these proteins in foetal fluids has been interpreted as reflecting physiological adaptations to increased foetal metabolic demands and the functional maturation of tissues, particularly during more advanced stages of pregnancy within the maternal‐foetal and extraembryonic interface (Vinogradov and Moens 2008). Thus, the identification of this protein in amniotic fluid reinforces the role of this compartment as a biologically active and dynamic medium that may contribute to the maintenance of physiological conditions conducive to foetal development within the extraembryonic gestational environment in sheep.

The study by Boy et al. (2019) in buffaloes provides a relevant comparative framework. The presence of shared proteins, such as albumin, alpha‐fetoprotein and apolipoproteins, indicates the existence of a conserved core of metabolic and transport functions during gestation in species with an epitheliochorial placenta. However, whereas proteins associated with antimicrobial defence predominate in buffaloes, our longitudinal approach in sheep revealed dynamic regulation of proteins linked to specific processes, such as proteolytic control (alpha‐2‐macroglobulin) and cytoskeletal remodelling (gelsolin). These differences reflect both physiological particularities between species and the experimental design, reinforcing the value of ruminants as models for comparative studies of the maternal–foetal and extraembryonic gestational microenvironment.

## 5 Conclusion

This study provides the first comparative and longitudinal proteomic characterization of allantoic and amniotic fluids throughout gestation in sheep, highlighting the dynamic nature of these compartments in maternal‐foetal and extraembryonic physiology. The progressive proteomic remodelling observed in both fluids reflects functional adaptations associated with foetal growth, tissue maturation and the maintenance of gestational homeostasis within the extraembryonic microenvironment. Rather than representing isolated protein changes, these alterations likely reflect coordinated physiological processes occurring at the maternal‐foetal interface, including changes in transport, metabolism and tissue remodelling. The identification of differentially abundant proteins involved in proteolytic regulation, tissue remodelling, redox balance, epithelial protection and metabolism supports the concept that foetal fluids act as biologically active compartments that may contribute to, rather than solely reflect, pregnancy maintenance within the maternal–foetal interface.

Moreover, comparisons with other ruminant studies reveal both conserved molecular functions and species‐specific adaptations, reinforcing the ovine model as a valuable platform for investigating the maternal‐foetal and extraembryonic gestational environment. However, these comparisons should be interpreted with caution, as differences in experimental design, gestational stages and analytical approaches may also contribute to the observed variability between studies. Collectively, these findings advance the understanding of gestational biology in ruminants and provide a foundation for future studies aimed at biomarker discovery and the development of strategies to improve reproductive efficiency and foetal health within the extraembryonic foetal microenvironment in animal production systems. Further functional validation will be necessary to confirm the specific roles of the proteins identified in this study.

## Author Contributions

**Viviane Maria Codognoto:** conceptualization, methodology, investigation, data curation, formal analysis, visualization, resources, project administration, writing – original draft, writing – review and editing. **Rogério Araújo de Almeida Filho:** conceptualization, methodology, investigation, data curation, formal analysis, visualization, resources, project administration, writing – original draft, writing – review and editing. **Marcos Gomides Carvalho:** data curation, formal analysis, writing – original draft, writing – review and editing. **Paula Zanin Rates:** data curation, writing – review and editing. **Flávio Augusto Lourencetti:** data curation, writing – review and editing. **Laiza Sartori de Camargo:** data curation, writing – review and editing. **Claudio Willian Delfes de Camargo:** data curation, writing – review and editing. **Juanita Valentina Guzmán Martínez:** data curation, writing – review and editing. **Luan Sitó da Silva:** writing – review and editing. **Eunice Oba:** conceptualization, methodology, investigation, funding acquisition, project administration, supervision, writing – original draft, writing – review and editing.

## Funding

This study was financed in part by the Coordenação de Aperfeiçoamento de Pessoal de Nível Superior—Brasil (CAPES)—Finance Code 001.

## Conflicts of Interest

The authors declare no conflicts of interest.

## Supporting information


**Figure S1:** Gene Ontology analysis of proteins identified in the allantoic fluid groups during early, mid and late gestation, considering molecular function, biological process and cellular component (https://bioinformatics.sdstate.edu/go/).

**Figure S2:** Gene Ontology analysis of proteins identified in the amniotic fluid groups during early, mid and late gestation, considering molecular function, biological process and cellular component (https://bioinformatics.sdstate.edu/go/).

## Data Availability

The data that support the findings of this study are available from the corresponding author upon reasonable request.
